# Health-Related Quality of Life and Return to Work after Surgery for Spinal Meningioma: A Population-Based Cohort Study

**DOI:** 10.3390/cancers13246371

**Published:** 2021-12-19

**Authors:** Jenny Pettersson-Segerlind, Ann-Christin von Vogelsang, Alexander Fletcher-Sandersjöö, Charles Tatter, Tiit Mathiesen, Erik Edström, Adrian Elmi-Terander

**Affiliations:** 1 Department of Neurosurgery, Karolinska University Hospital, 171 64 Stockholm, Sweden; jenny.pettersson.segerlind@ki.se (J.P.-S.); ann-christin.von-vogelsang@ki.se (A.-C.v.V.); alexander.fletcher-sandersjoo@ki.se (A.F.-S.); charles.tatter@ki.se (C.T.); erik.edstrom.1@ki.se (E.E.); 2 Department of Clinical Neuroscience, Karolinska Institutet, 171 77 Stockholm, Sweden; tiit.illimar.mathiesen@regionh.dk; 3 Department of Neurosurgery, Rigshospitalet, Institute of Clinical Medicine, University of Copenhagen, 2100 Copenhagen, Denmark

**Keywords:** spinal meningioma, neurosurgery, patient-reported outcomes, health-related quality of life, return to work

## Abstract

**Simple Summary:**

Spinal meningioma is the most common primary intradural spinal tumor. Although histologically benign, the tumors often cause neurological deficits. Health-related quality of life (HRQoL) is defined as the aspects of quality of life which are most affected by ill health and is a measure of self-perceived health status. Despite many studies evaluating the neurological outcome after surgery for spinal meningiomas, no study has been concerned with the HRQoL and frequency of return to work. In this population-based cohort study, we reviewed 84 cases of surgically treated spinal meningiomas, with a mean follow-up of 8.7 years, to assess their HRQoL compared to a sample of the general population. We found that HRQoL after surgery was equal to the normal population, and the frequency of return to work was 100%, often within three months of surgery. Thus, surgical treatment of spinal meningiomas should not be considered a threat to long-term quality of life.

**Abstract:**

Spinal meningiomas are the most common primary spinal intradural tumor. This study aimed to assess Health-related quality of life (HRQoL) and the frequency of return to work in patients surgically treated for spinal meningiomas, in comparison to the general population. Variables were collected from patient charts, EQ-5D-3L, and study specific questionnaires. Patients who had been operated between 2005–2017 were identified in a previous study and those alive in 2020 (104 of 129) were asked to participate. Eighty-four patients (80.8%) with a mean follow-up of 8.7 years, responded and were included. Data was compared to the Stockholm Public Health Survey 2006, a cross-sectional survey of a representative sample of the general population. Analysis for potential non-response bias showed no significant differences. Women in the meningioma sample scored more problems than men with regards to mobility (*p* = 0.048). There were no significant differences concerning EQ-5D_index_ (*p* = 0.325) or EQ_VAS_ (*p* = 0.116). The correlation between follow-up time and EQ-5D_index_ was low (*r* = 0.167). When comparing HRQoL to the general population sample, no significant differences were found within the EQ-5D-3L dimensions, EQ-5D_index_ or EQVAS. Those who postoperatively scored 3–5 on mMCs scored significantly more problems in the EQ-5D-3L dimension mobility (*p* = 0.023). Before surgery, 41 (48.8%) of the spinal meningioma patients were working and after surgery all returned to work, the majority within three months. Seventy-eight (96%) of the patients would accept surgery for the same diagnosis if asked today. We conclude that surgery for spinal meningiomas is associated with good long-term HRQoL and a high frequency of return to work.

## 1. Introduction

Health related quality of life (HRQoL) is defined as the aspects of quality of life which are most affected by ill health and is a measure of self-perceived health status [1]. Quality of life is also reflected in an individual’s ability to work while the possibility to return to work after surgery has a great impact on quality of life [2,3,4,5,6,7]. In previous HRQoL studies on Swedish populations, the frequencies of reported health problems increased with age and women generally reported more health problems than men [8,9].

Spinal meningiomas are benign, slow growing tumors. They comprise 25–45% of intradural spinal tumors and have an age adjusted incidence of 3.3 per million yearly [10]. The majority are WHO grade 1, with higher WHO grades reported in 1.5–8.5% of cases [11,12,13,14,15]. These tumors are not life threatening and the vast majority of operated patients improve after surgery [16]. Large meningiomas can compress the spinal cord or nerves and consequently affect neurological function and the main preoperative finding is motor deficit [12]. Surgery is performed to halt neurological deterioration but also often improves the neurological condition and the associated patient reported outcomes such as pain [12,17].

While several studies have reported data on the neurological outcomes after surgery for spinal meningiomas, the available literature on HRQoL is limited to small studies of intradural tumors. A study by Viereck et al. on 44 intradural extramedullary tumors (14 meningiomas) showed significant and lasting improvements in HRQoL after surgery [18]. Similarly, Newman et al. found lasting improvements in patient reported outcomes after surgery in 57 patients (18 meningiomas) with benign spinal extramedullary tumors [17]. No studies have reported on return to work after spinal meningioma surgery.

In contrast, quality of life and return to work have been extensively studied in cranial meningiomas [19,20,21,22]. After surgery, anxiety, depression, and fatigue are common [23,24,25,26,27,28]. In the long term, quality of life may also be negatively affected by tumor recurrence, which is unexpectedly high in cranial meningiomas but not in spinal meningiomas [12,29,30,31,32]. Moreover, reportedly asymptomatic patients with cranial meningiomas, who have not undergone surgery, also have a high incidence of psychiatric complaints that are likely to impact quality of life [33,34].

Studies on return to work have indicated that 17–33% of the patients operated for cranial meningioma were unable to return to work [26,27,28]. A recent Swedish study on patterns of sick leave prior to surgery and return to work up to two years after surgery in patients with intracranial meningiomas, showed that 79.0% of the patients were working full time one year prior to surgery. That number declined to 49.3% at one year and was only 57.3%, two years after surgery, while the level of employment for controls remained constant at 84% to 86% [35].

It is reasonable to discuss spinal meningiomas in comparison to meningiomas located intracranially or to other spinal pathologies. On the one hand, a comparison to cranial meningiomas can be justified based on the histopathological similarities and the fact that patients are treated for a neoplastic disease. On the other hand, a comparison to spinal disorders can be justified based on similarities of symptoms and surgical approaches. In that context, lumbar spinal stenosis (LSS) and its surgical treatment share many features with spinal meningiomas. Multiple reports on HRQoL in LSS showed a lower HRQoL compared to the general population [36], that women reported lower HRQoL than men [37], and that there was an overall improvement in HRQoL after surgery [38,39]. Still, 60–75% of patients operated for LSS did not return to work [40,41].

The objective of this study was to explore long-term HRQoL and return to work in a consecutive cohort of patients surgically treated for spinal meningiomas, who were previously investigated for neurological outcomes after surgery [12].

## 2. Materials and Methods

This study has a comparative approach, comparing HRQoL data from a spinal meningioma sample with a general population sample. Data on HRQoL were self-reported on EQ-5D-3L, while data on employment, sick leave, and return to work were extracted from a study specific questionnaire.

### 2.1. Samples

#### 2.1.1. Spinal Meningioma Sample

**Figure 1:**
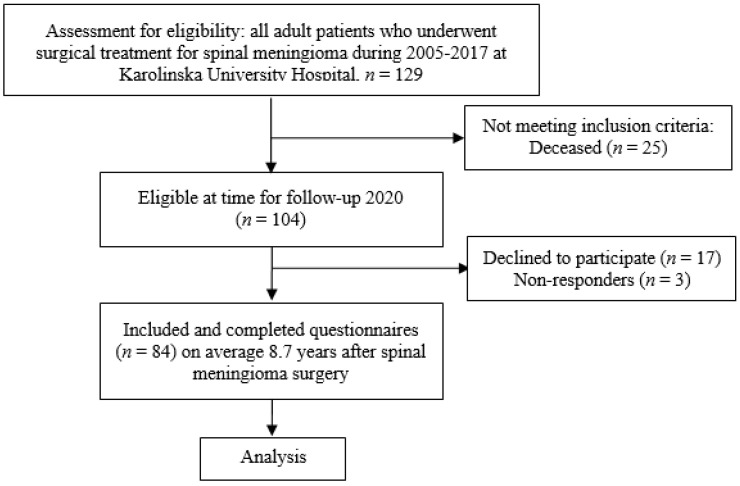
Flowchart of the patient inclusion process.

All adult (≥18 years) patients operated for a spinal meningioma at the Karolinska University Hospital over a period of 13 years (2005–2017), were identified in a previous study including 129 patients [12]. Of these 129 patients, 104 were still alive in 2020 and were contacted with a request for participation in this follow-up study. A total of 20 patients declined to participate or did not respond. Thus, 84 spinal meningioma patients were included in the study (80.8% of eligible patients; Figure 1).

#### 2.1.2. General Population Sample

Data from the Stockholm Public Health Survey 2006 was used as the comparative general population sample. This was a cross-sectional survey of a representative sample of the general population in Stockholm County. A self-reported postal questionnaire, including the EQ-5D-3L, was sent to 57,000 persons aged 18–84 years, with a response rate of 61%. The occurrence of long-standing illness was self-reported with a specific question including long-term illness, after-effects from an accident, disability or other ailments [9]. Raw data was obtained, and for each one of the 84 spinal meningioma patients, three control subjects were randomly selected and individually matched by sex and age, no selection concerning long-standing illness was conducted before the randomization. Thus, 252 individuals were included in the general population sample, intended to mirror the population in the Stockholm region. Seven women in the spinal meningioma sample were older (aged 85 to 90 years) than the respondents in the Stockholm Public Health Survey; their controls were therefore matched with the oldest controls, aged 84 years old.

### 2.2. Measures

#### 2.2.1. EQ-5D-3L

The EQ-5D-3L measures HRQoL and consists of two parts. The first part is a descriptive system in which the respondents classify their health in 5 dimensions (mobility, self-care, usual activities, pain/discomfort, and anxiety/depression) with 3 severity levels (no problems, moderate problems, or severe problems) [42]. The response value of each dimension is combined into a 5-digit value representing a corresponding health state, which can be indexed into a single overall HRQoL value, EQ-5D_index_, where 0 represents dead and 1 represents full health. However, some health states are on a group level considered worse than death and are assigned negative values [43]. A total of 243 health states can be elicited from the descriptive system. In this study, the United Kingdom (UK) value set was used to calculate the EQ-5D_index_ [44]. The second part is the EQ visual analogue scale (EQ_VAS_), where the respondents rate their current health between 0 (worst imaginable health) and 100 (best imaginable health).

#### 2.2.2. Study-Specific Questionnaire

A study specific questionnaire with multiple choice questions was designed. The questions regarded neurological symptoms (motor and sensory) in the upper and lower extremities as well as balance and incontinence and how these symptoms had changed following surgery. The patients were also asked whether they would accept the same surgery had it been offered to them today. Questions were also asked about Charlson comorbidity index components, problems with arthritis, and current medication. The final part concerned employment, sick-leave, and return to work after surgery.

#### 2.2.3. Variables Retrieved from Electronic Medical Records

Pre-operative data regarding age, sex, American Society of Anesthesiologists (ASA) class, prior radiotherapy, prior spinal surgery, neurological symptoms, modified McCormick Scale (mMCs), tumor location, and postoperative data regarding time from diagnosis to surgery, laminectomy range, Simpson grade, MIB1-index, World Health Organization (WHO) grade, adjuvant treatment, postoperative complications, follow-up time, long-term tumor growth and/or recurrence, long-term neurological symptoms, and change in mMCs, were collected as part of a previous study [12].

### 2.3. Data Analysis

A non-response analysis was conducted, and the following variables were analyzed; age at surgery, sex, employment status before surgery, employment status 0–12 months after surgery, modified McCormick scale (mMCs) [45] before and after surgery.

To compare pre- and postoperative mMCs assessments in the *n* = 84 included patients, the related samples marginal homogeneity test was used.

The EQ-5D-3L data were compared between the spinal meningioma sample and the general population sample. Furthermore, when analyzing EQ-5D-3L data in the spinal meningioma sample, participants neurologically intact or with mild sensory/motor deficits postoperatively (mMCs grade 1–2) were compared to participants with postoperative moderate or severe sensory/motor deficits (mMCS grade 3–5). Descriptive statistics for the demographic and study-specific variables, EQ-5D dimensions, EQ-5D_index_, and EQ_VAS_ were calculated. To analyze differences between groups, the chi-squared test, Fisher’s exact test, and the t-test were used. Moderate and severe levels on EQ-5D dimensions were collapsed before performing the chi-square statistics. The Pearson correlation coefficient was used to examine associations between follow-up time and EQ-5D_index_. Statistical significance was set at *p* < 0.05.

### 2.4. Ethical Considerations

A signed informed consent was obtained from each participant in the spinal meningioma sample. Data from the Stockholm Public Health Survey was based on individuals who gave informed consent to participate and was anonymized when obtained. The study was approved by the Regional Ethical Review Board (registration number 2016/1708-31/4 and the National Ethical Review Authority (registration numbers 2020-00192 and 2021-03623).

## 3. Results

### 3.1. Characteristics of Samples

There were no significant differences between the responding patients and those who did not respond concerning age at surgery (61.9 ± 11.6 versus 58.9 ± 18.2 years; *p* = 0.307), or sex (84.5%, *n* = 71 versus 75.0%, *n* = 15, women; *p* = 0.240). When analyzing employment status before and after surgery, there were no differences before surgery between included patients and those who did not respond (*p* = 0.112), but up to 12 months after surgery there was a significantly higher proportion in the non-response group that were on sick-leave or having sickness pension (4.8%, *n* = 4 versus 25.0%, *n* = 5; *p* = 0.004). Notably, four of these five patients were already on sick leave preoperatively. There were no significant differences between groups concerning preoperative mMCs (*p* = 0.454) or postoperative mMCs (*p* = 0.261). In the 84 included patients, mMCs improved significantly on a group level (*p* < 0.001), where *n* = 39 improved, *n* = 44 had unchanged mMCs (of whom 22 were mMCs 1 prior to surgery and could not improve) and *n* = 1 worsened from mMCs 2 to 3. The average age at follow-up was 70.6 years in the spinal meningioma sample, versus 70.3 years in the general population sample. Most spinal meningioma patients had thoracic meningiomas and underwent 2 to 3 level laminectomies (Table 1).

### 3.2. HRQoL in the Spinal Meningioma Sample

**Figure 2:**
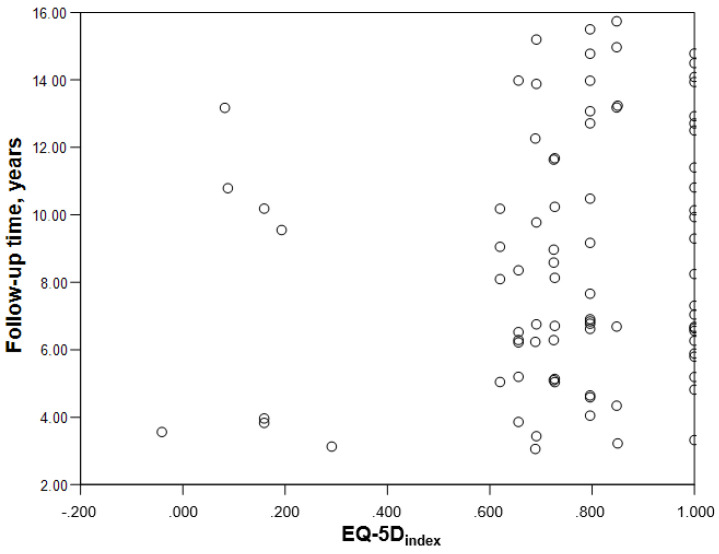
Scatterplot demonstrating lack of correlation between the time from surgery and the EQ-5D_index_.

The women in the spinal meningioma sample scored worse than the men in all dimensions of the descriptive system (Table 2), but the differences were only significant in the dimension mobility (*p* = 0.048). There were no significant differences concerning EQ- 5D_index_ (*p* = 0.325) or EQ_VAS_ (*p* = 0.116). The correlation between follow-up time and EQ-5D_index_ was low, *r* = 0.167 (Figure 2).

### 3.3. Comparison of HRQoL between Spinal Meningioma Sample and General Population Sample

Of the possible 243 health profiles that can be elicited from the EQ-5D descriptive system, a total of 16 profiles were reported by the participants in the spinal meningioma sample compared to 31 profiles in the general population sample. One participant in the spinal meningioma sample had an EQ-5D_index_ value below zero, −0.041 (representing 2, 1, 1, 3, 3 in the descriptive system). In the general population sample three participants had an EQ-5D_index_ value below zero; the lowest score was −0.095, representing 3, 3, 3, 2, 1 in the descriptive system.

Full health (i.e., a score of 1, 1, 1, 1, 1 in the descriptive system) was reported by 28.6% (*n* = 24) in the spinal meningioma sample, and 28.2% (*n* = 71) in the general population sample. Best imaginable health (i.e., a score of 100 on EQ_VAS_) was reported by one participant (1.2%) in the spinal meningioma sample, and 14 (5.6%) in the general population sample. The most frequent EQ_VAS_ score in both samples was 90, reported by 14 (15.5%) in the spinal meningioma sample and 40 (15.9%) in the general population sample.

No significant differences were found within the EQ-5D-3L dimensions, EQ-5D_index_ or EQ_VAS_ between the samples (Table 2).

### 3.4. Comparison of HRQoL between Participants Differing in mMCs Grade

**Figure 3:**
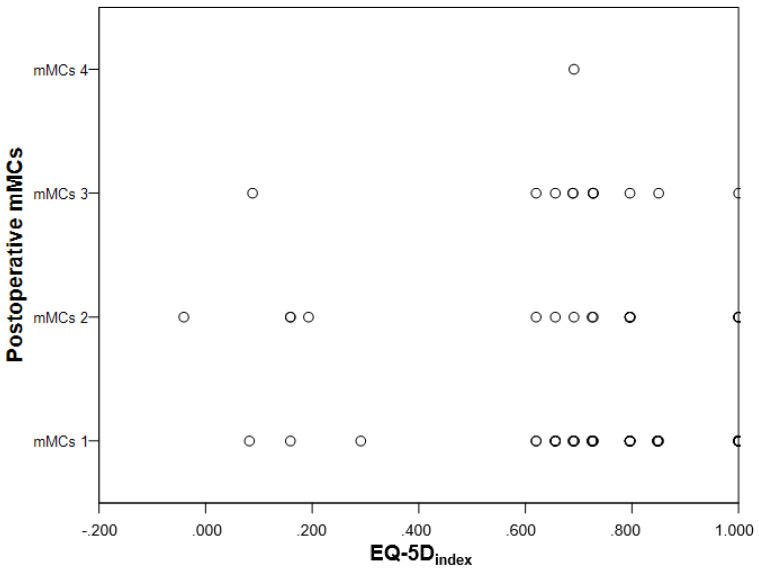
Scatterplot showing lack of correlation between the postoperative mMCs grade and EQ-5D_index_.

Participants that were mMCs grade 3–5 postoperatively, scored more problems in the EQ-5D-3L dimension mobility (*p* = 0.023). There were no significant differences in any of the other four EQ-5D-3L dimensions, EQ_VAS_ or EQ-5D_index_ (Table 3, Figure 3).

### 3.5. Employment Status and Return to Work after Spinal Meningioma Surgery

Before surgery, a total of 41 (48.8%) of the spinal meningioma patients were working full or part time, and all of them returned to work after surgery, all but one to the same workplace. The majority returned to work within three months. Five of the nine patients who before surgery were on sick leave or had sickness pension received old age pension after surgery (Table 4).

### 3.6. Comorbidity

The Charlson comorbidity index was 0–2 in 82.2% of the patients (Table 1). Arthritis, asked as an additional question, was present among 44 (53%) patients. Of these patients 19 (43%) reported mobility disturbances in their EQ-5D. One patient did not answer this question.

### 3.7. Medication

Thirty-one (37%) patients responded “yes” to the use of medication for pain or spasticity. In this group, all used NSAID or paracetamol, five (6%) used morphine derivates, six (7%) used neuralgic pain relief medication (gabapentin or pregabalin) and one patient (1%) anti spastic medication (Baclofen).

### 3.8. Remaining Symptoms and Patient Reported Outcome

Sixty-seven (80%) of the patients reported at least one remaining symptom in the study specific questionnaire (Table 1). Fifty-three patients (63%) reported improvement postoperatively, 21 (25%) reported no change, and eight (10%) reported worsening in their self-assessed neurological symptoms. Two patients (2%) did not answer this question. Seventy-eight (96%) patients responded that they would accept surgery for the same diagnosis if asked today. Three patients did not answer this question and another three patients responded that they would not accept surgery if asked today. The postoperative neurological status was unchanged for these three patients. Among them, one had severe and persistent musculoskeletal neck pain, not related to the tumor, and two developed neuralgic pain. All three had a low EQ_VAS_ (40–50).

The frequency of peri- (one) and postoperative (seven) complications was too low to allow statistical analysis. Five of the postoperative complications had resolved at short-term follow-up. The remaining perioperative (spinal cord injury) and postoperative complications (myocardial infarction and asymptomatic kyphosis) were not likely to alter the overall results.

## 4. Discussion

In this study, the HRQoL of patients with spinal meningiomas at long-term follow up did not differ from that of the general population. The majority of meningioma patients were women (85.7%), but no differences between the sexes could be seen. The mean EQ_vas_ value was 74.0—slightly higher than the mean value of 71.7 in the general population sample, although not significant. Frequency of pain was not different between patients and controls while a slightly higher proportion of the meningioma patients reported moderate mobility problems. Interestingly, McCormick grade or time after surgery (ranging 4–15 years) did not correlate with the overall HRQoL (EQ-5D_index_). The frequency of comorbidities in our cohort was very low, 82.2% scoring ≤2 on the Charlson comorbidity index, even though assessed 4–15 years after surgery. All patients who were working before surgery returned to work, mostly within 3 months and all within a year. The number on sick leave decreased by 6% and only 12 patients (14%) needed prescription drugs for tumor related pain.

Few studies have addressed quality of life after surgery for spinal meningiomas. In a mixed group of different intradural spinal tumors, patient-reported outcome measures indicated that pain was the predominant preoperative cause of suffering. Postoperatively, pain was reduced, and patients experienced significant improvements regarding general activity, mood, walking ability, ability to participate in normal work, quality of relations, quality of sleep, and enjoyment of life [17]. Another study showed significant and lasting improvements in HRQoL after surgery for spinal intradural tumors [18]. These findings are in accordance with our results. In contrast to the schwannomas and myxopapillary ependymomas included in these studies, meningiomas affect elderly and typically produce dramatic spinal cord compression. Thus, good surgical outcomes cannot be taken for granted. The spinal meningioma patients recovered and returned to previous activities with a quality of life not different to that of the general population, suggesting that surgery could relieve symptoms without adding morbidity and patients could view themselves as cured.

Due to the scarcity of HRQoL data on spinal meningiomas, we chose to make the comparison to cranial meningiomas and LSS. The long-term quality of life after surgery for cranial meningiomas has been extensively investigated. Despite the same histopathological diagnosis, the impact on quality of life is markedly different. The initial symptoms for spinal meningioma include pain and neurological deficits reflecting compression of the spinal cord or nerves. In this respect, patients with spinal meningioma resemble those with LSS. Arguably, cranial and spinal surgery carry different risks and implications for health in the long-term. However, also in spinal surgery, here represented by LSS, quality of life remains affected and only a minority of patients return to work after surgery [41,46]. Thus, the results of this study serve to distinguish spinal meningiomas as a disease with a surgical cure and good long-term HRQoL.

HRQoL in cranial meningiomas is typically improved by tumor resection while long-term follow-up shows persistent reduced HRQoL in comparison to healthy controls [22]. Decreased HRQoL after cranial meningioma surgery appears in part to be associated with epilepsy, cognitive deficit, and tumor recurrence. However, even patients with asymptomatic meningiomas, who have not undergone surgery, have severely affected health [33]. Psychological factors including anxiety and depression are common in patients with cranial meningiomas [33,34,47,48]. This is not the case for spinal meningiomas which do not cause epilepsy or affect cognition and rarely recur [12,49]. Moreover, the psychological distress related to a tumor diagnosis is well documented for cranial but not spinal meningiomas [48].

Compared to cranial meningiomas, LSS and spinal meningiomas have similarities in symptoms and surgical approaches. Meningiomas may initially be misdiagnosed as LSS [50,51,52]. Battié et al. compared a sample of 245 patients with LSS to a sample of 7489 from the general population. Using the Health Utilities Index Mark 3 (HUI3), they found that the mean unadjusted overall scores were 0.60 for LSS and 0.85 for the general population group (1 = perfect health). Even after adjustment for age and sex, large differences in HRQoL remained between the groups, and the frequency of comorbidities was significantly higher in the LSS group [36]. Kobayashi et al., found that the HRQoL of women with LSS was lower than men with the same diagnosis [37]. HRQoL was significantly improved following surgery [38,39].

Truszczyńska et al., studied 58 patients with LSS with an even sex distribution. In that cohort, only 13 (22.3%) returned to work after surgery. Half of the patients intended to apply for disability pension, 28% considered themselves unfit for work, and 38% did not feel like working again. The domain scores for physical health, psychological health, social relations with friends and family or at work ranged from 59 to 61 on the WHOQOL-BREF, transformed to a 0–100 scale making it comparable to EQ_vas_. However, the quality of life of the patients who did return to work was similar to that of a healthy normal population [41]. Herno et al., compared the ability to work of 185 women and 254 men after LSS surgery and found that, excluding retired patients, only 37% of the women and 41% of the men returned to work. None of the patients who had retired before their operation returned to work after surgery [46].

In summary, this study provides the first report on the HRQoL and return to work for a spinal meningioma cohort compared to a general population sample. The data provides new insights regarding the overall health, comorbidities, and outcomes of surgically treated spinal meningioma patients, where previous data is lacking. The long-term, post-surgery, HRQoL of this cohort is equal to that of the general population and much better than that of both cranial meningiomas and LSS. The patients are satisfied with their treatment and almost all would accept the same treatment if offered today.

### Methodological Considerations, Strengths, and Limitations

This study is retrospective and provides a snapshot 4–15 years after surgery. The general population sample data was collected in 2006, before the spinal meningioma sample data. Analysis of repeated population surveys using the EQ-5D-3L during 1998 and 2002 showed deteriorating health over time [8], but later population surveys were not published.

Strengths of the study include the population-based cohort of patients and controls, and the homogenous and relatively large sample of spinal meningioma patients. Surgeon’s bias was limited by the institutional routine which mandates that all referred cases of spinal meningioma with manifest or imminent spinal cord compression should be offered surgery. Furthermore, in the spinal meningioma sample the response rate was high (81%), as was the granularity of the collected data. The general population sample could be retrieved from the same Swedish region and importantly, since HRQoL differs between ages and sexes [8,9], the spinal meningioma patients were matched by age and sex. Moreover, the general population sample also included respondents with long-standing illness, not only those with perfect health.

Of the 129 consecutive patients, 25 had died and 20 of 104 eligible patients did not participate. While we cannot exclude some selection bias, most comparable parameters such as age, sex and pre/postoperative McCormick grades were not statistically different between those who responded to the questionnaire and those who did not. A larger proportion of patients on sick leave or old-age pension chose not to respond. However, even extreme responses from the five out of nine on sick leave would not significantly alter the results. Moreover, the matched controls were selected from a population sample with a 61% response rate which may also have given an overly favorable assessment of HRQOL among the normal population. We conclude that our data is representative and has external validity for surgically treated spinal meningioma patients. In our cohort of 84 patients, outcomes were related to time to surgery and degree of spinal cord compression; impact on HRQoL from tumor characteristics, postoperative deformity [53] or tumor recurrence was not detectable. Yet, a larger study and longer follow-up may reveal smaller associations with such objective parameters.

Another limitation is that the study-specific questionnaire was not validated. However, the questions mainly concerned diagnosis, treatment and employment status and were considered to have face validity.

## 5. Conclusions

In support of previous reports, surgery for spinal meningioma was found to be safe, with few complications and overall good outcomes, even in the elderly. Despite a high median age at surgery, the HRQoL at long-term follow up was equivalent to a matched sample of the general population. The spinal meningioma sample showed few comorbidities, few remaining symptoms, and limited use of pain medication. All who were able to work preoperatively returned to work within one year postoperatively. Based on the findings of this study, and a previous report on the same cohort, we argue that surgery should be considered for all patients with a spinal meningioma.

## Figures and Tables

**Table 1:** Characteristics of the spinal meningioma sample.

Variable	Value (*n* = 84)
Male sex	12 (14.3%)
Age (years) at follow-up (range)	70.6 ± 10.6 (42–92)
Arthritis	44 (52.4%)
Time (years) from surgery to follow-up (range)	8.7 ± 3.7 (3.1–15.7)
Tumor location *	
Cervical	29 (34.5%)
Thoracic	55 (65.5%)
Charlson Comorbidity Index	
0	25 (29.8%)
1	24 (28.6%)
2	20 (23.8%)
3	8 (9.5%)
4	5 (6.0%)
5	1 (1.2%)
Missing data	1 (1.2%)
Laminectomy range (levels)	
1	3 (3.6%)
2	40 (47.6%)
3	31 (36.9%)
4	8 (9.5%)
5	1 (1.2%)
6	1 (1.2%)
Modified McCormick scale before surgery	
mMCs 1, neurologically intact	22 (26.2%)
mMCs 2, mild motor or sensory deficits, functional independence	37 (44.0%)
mMCs 3, moderate deficits, limited function, independent	22 (26.2%)
mMCs 4, severe deficits, limited function, dependent	3 (3.6%)
mMCs 5, paraplegia or quadriplegia	0 (0.0%)
Modified McCormick scale after surgery	
mMCs 1, neurologically intact	53 (63.1%)
mMCs 2, mild motor or sensory deficits, functional independence	19 (22.6%)
mMCs 3, moderate deficits, limited function, independent	11 (13.1%)
mMCs 4, severe deficits, limited function, dependent	1 (1.2%)
mMCs 5, paraplegia or quadriplegia	0 (0.0%)
Remaining symptoms after surgery	
Sensory symptoms upper extremities	22 (26.2%)
Sensory symptoms lower extremities	24 (28.6%)
Motor symptoms upper extremities	29 (34.5%)
Motor symptoms lower extremities	38 (45.2%)
Balance problems	47 (56.0%)
Incontinence	23 (27.4%)

* Tumor location is defined by the uppermost laminectomy level. Data is presented as number (proportion) or mean ± SD [range]. Abbreviations: mMCs = modified McCormick Scale.

**Table 2:** Percentage (number) of participants reporting no, moderate or severe problems in EQ-5D dimensions, EQ-5D_index_, and EQ_VAS_, spinal meningioma sample and general population sample.

	Total					Men					Women				
EQ-5D Dimensions	Spinal Meningioma *n* = 84		General Population *n* = 252		*p* ^1,2^	Spinal Meningioma *n* = 12		General Population *n* = 36		*p* ^1,2^	Spinal Meningioma *n* = 72		General Population *n* = 216		*p* ^1,2^
	%	*n*	%	*n*		%	*n*	%	*n*		%	*n*	%	*n*	
**Mobility**					0.221					0.414					0.108
No problems	63.3	54	71.4	180		91.7	11	75.0	27		59.7	43	70.8	153	
Moderate problems	35.7	30	28.2	71		8.3	1	25.0	9		40.3	29	28.7	61	
Severe problems	0.0	0	0.4	1		0.0	0	0.0	0		0.0	0	0.5	1	
**Self-care**					0.198					ns					0.196
No problems	98.8	83	95.2	240		100.0	12	100.0	36		98.6	71	94.4	204	
Moderate problems	1.2	1	3.6	9		0.0	0	0.0	0		1.4	1	4.2	9	
Severe problems	0.0	0	1.2	3		0.0	0	0.0	0		0.0	0	1.4	3	
**Usual activities**					0.878					1.000					1.000
No problems	79.8	67	78.6	198		91.7	11	88.9	32		77.8	56	76.9	166	
Moderate problems	20.2	17	17.5	44		8.3	1	11.1	4		22.2	16	18.5	40	
Severe problems	0.0	0	4.0	10		0.0	0	0.0	0		0.0	0	4.6	10	
**Pain/discomfort**					0.896					0.341					0.886
No problems	36.9	31	36.1	91		58.3	7	41.7	15		33.3	24	35.2	76	
Moderate problems	56.0	47	56.3	142		41.7	5	52.8	19		58.3	42	56.9	123	
Severe problems	7.1	6	7.5	19		0.0	0	5.6	2		8.3	6	7.9	17	
**Anxiety/depression**					0.589					1.000					0.571
No problems	65.5	55	69.0	174		83.3	10	86.1	31		62.5	45	66.2	143	
Moderate problems	31.0	26	29.8	75		8.3	1	13.9	5		34.7	25	32.4	70	
Severe problems	3.6	3	1.2	3		8.3	1	0.0	0		2.8	2	1.4	3	
EQ-5D_index_ mean (±SD)	0.76 (±0.24)		0.75 (±0.25)		0.928	0.86 (±0.22)		0.82 (±0.21)		0.598	0.74 (±0.24)		0.74 (±0.26)		0.932
EQ_VAS_ mean (±SD)	74.0 (±17.9)		71.7 (±21.7)		0.392	78.7 (±15.4)		78.2 (±17.4)		0.934	73.1 (±18.3) ^3^		70.6 (±22.1)		0.390

Data is presented as number (proportion) or mean (SD). ^1^. Differences between spinal meningioma sample and general population sample. ^2^. Moderate and severe levels in EQ-5D dimensions collapsed before Chi-square analysis. ^3^. Missing values, *n* = 4.

**Table 3:** Comparison of participants with mMCs grade 1–2 to mMCs 3–5 regarding EQ-5D dimensions, EQ-5D_index_ and EQ_VAS_.

EQ-5D Dimensions	mMCs 1–2 *n* = 72		mMCs 3–5 *n* = 12		*p* ^1^
	%	n	%	n	
**Mobility**					0.023
No problems	69.4	50	33.3	4	
Moderate problems	30.6	22	66.7	8	
Severe problems	0.0	0	0.0	0	
**Self-care**					1.000
No problems	98.6	71	100.0	12	
Moderate problems	1.4	1	0.0	0	
Severe problems	0.0	0	0.0	0	
**Usual activities**					0.060
No problems	83.3	60	58.3	7	
Moderate problems	16.7	12	41.7	5	
Severe problems	0.0	0	0.0	0	
**Pain/discomfort**					0.196
No problems	40.3	29	16.7	2	
Moderate problems	52.8	38	75.0	9	
Severe problems	6.9	5	8.3	1	
**Anxiety/depression**					0.744
No problems	66.7	48	58.3	7	
Moderate problems	29.2	21	41.7	5	
Severe problems	4.2	3	0.0	0	
EQ-5D_index_ mean (±SD)	0.77 (±0.25)		0.69 (±0.21)		0.293
EQ_VAS_ mean (±SD)	74.0 (±18.1) ^2^		73.4 (±17.3) ^3^		0.908

Data is presented as number (proportion) or mean (±SD). ^1^ Moderate and severe levels in EQ-5D dimensions collapsed before Chi-square analysis. ^2^ Missing values, *n* = 3. ^3^ Missing values, *n* = 1.

**Table 4:** Employment status and return to work.

Employment Status and Timing for Return to Work	*n* (%)
**Employment status before surgery, *n* = 84**	
Full time	35 (41.7%)
Part time work	6 (7.1%)
Full time sick leave	3 (3.6%)
Sickness pension	6 (7.1%)
Old-age pension	34 (40.5%)
**Employment status after surgery, *n* = 84**	
Full time	35 (41.7%)
Part time work	6 (7.1%)
Full time sick leave	1 (1.2%)
Sickness pension	3 (3.6%)
Old-age pension	39 (46.4%)
**Timing for return to work, *n* = 41**	
Return to full time work	
Within <3 months	20 (48.9%)
From 3 to <6 months	11 (26.8%)
From 6 to <12 months	4 (9.6%)
≥12 months	0 (0.0%)
Return to part time work	
Within <3 months	2 (4.9%)
From 3 to <6 months	2 (4.9%)
From 6 to <12 months	1 (2.4%)
≥12 months	1 (2.4%)

Data is presented as number (proportion).

## Data Availability

Data is available from the corresponding author upon reasonable request.

## Abbreviations

ASA	American Society of Anesthesiologists
HRQoL	Health-Related Quality of Life
LSS	lumbar spinal stenosis
mMCs	modified McCormick scale
MRI	magnetic resonance imaging
OR	odds ratio
WHO	World Health Organization
